# Arrhythmias related to antipsychotics and antidepressants: an analysis of the summaries of product characteristics of original products approved in Germany

**DOI:** 10.1007/s00228-020-03049-x

**Published:** 2020-11-23

**Authors:** Mohamed Elsayed, Emaad Abdel-kahaar, Maximilian Gahr, Bernhard J. Connemann, Michael Denkinger, Carlos Schönfeldt-Lecuona

**Affiliations:** 1 Department of Psychiatry and Psychotherapy III, University of Ulm, Leimgrubenweg 12-14, 89075 Ulm, Germany; 2 Institute of Pharmacology of Natural Products and Clinical Pharmacology, University of Ulm, Ulm, Germany; 3 Department of Pharmacology, Qena Faculty of Medicine, South Valley University, Qena, Egypt; 4 Agaplesion Bethesda Clinic, Geriatric Research Unit Ulm University, Ulm, Germany; 5 Geriatric Center Ulm/Alb-Donau, Ulm, Germany

**Keywords:** Arrhythmias, SmPCs, QTc prolongation, Sudden cardiac death, Psychiatric drug

## Abstract

**Purpose:**

Most psychiatric drugs, such as antidepressants (AD) and antipsychotics (AP), may cause cardiac adverse events (CAE). We used summaries of product characteristics (SmPC) for assessing the likelihood of AD and AP to cause CAE.

**Methods:**

We identified all original medicinal products (OMP) of AD and AP approved in Germany. We searched for their SmPCs using the online services of PharmaNet.Bund, Gelbe liste®, Rote Liste®, Fachinfo-Service®, and via manufacturer contact. We extracted frequencies of reported CAE (QT prolongation, Torsade de Pointes tachycardia, and ventricular arrhythmia) and performed a risk assessment.

**Results:**

We obtained the SmPCs of 24 AD and 26 AP identified as OMP. Comparably high reported frequencies regarding QT prolongation were found for Invega® (paliperidone), Serdolect® (sertindole) (≥ 1/100 and < 1/10), and Zoloft® (sertraline) (≥ 1/10.000 and < 1/1000); regarding Torsade de Pointes tachycardia were found for Serdolect® (≥ 1/1000 to < 1/100), Zoloft®, and Trevilor® (venlafaxine) (≥ 1/10.000 and < 1/1000); regarding ventricular tachycardia for Solian® (amisulpride), Xomolix® (droperidol), Zyprexa® (olanzapine), and Trevilor® (≥ 1/10.000 and < 1/1000).

**Conclusion:**

The risk and frequency of CAE, as reported in the SmPCs, varied significantly among substances and between groups. There are more reports for AP than AD. The AP with the most frequently reported CAE (QT prolongation and Torsade de Pointes tachycardia) was Serdolect®; for AD, Zoloft® (QT prolongation, Torsade de Pointes tachycardia) and Trevilor® (Torsade de Pointes tachycardia and ventricular tachycardia) carried a higher cardiac risk.

## Introduction

Cardiac adverse events (CAE) can potentially arise from both psychiatric and non-psychiatric drugs [1]. They range from tachycardia to potentially lethal ventricular arrhythmias, sudden cardiac arrest, and sudden cardiac death [1]. In psychiatry, much attention has been paid to evaluate the risk of psychiatric drugs of all substance groups to cause arrhythmias and to describe drug-induced disturbances of cardiac repolarization, such as prolongations of the QT-interval [1]. Psychiatric drugs, such antipsychotics (AP) and antidepressants (AD), are very frequently prescribed in the pharmacological treatment of psychiatric and other disorders [1]. Since 2005, a “thorough QT study” (TQT) is required to evaluate the pro-arrhythmic tendency of new drugs [2]. However, the introduction to the market of most of the currently used medications was before the implementation of TQT studies [2]. Furthermore, a precise single-substance TQT study cannot exclude pro-arrhythmia, e.g., when a drug is used in combination with other medicines, or when comorbid heart disease is present [2]. Some older substances that were used over the years worldwide, whose approval studies were not so extensive, such as the AP thioridazine, were withdrawn from the market due to such complications after analyses of post-marketing surveillance [1, 3], or safety measures have been added to their license (such as periodical controlling for QT interval) regulate the use, e.g., Serdolect® (sertindole) [3]. Despite these efforts in safety measures, there is an ongoing debate about the relative cardiac risk of every single substance in each psychiatric drug group, especially concerning QT prolongation and cardiac arrhythmias [4].

Previous studies have focused mainly on AP and their potential to cause QT prolongation [5]. Several studies have also created rankings of psychiatric drugs according to their risk of causing QTc-prolongation. On the other hand, in other substance classes, e.g., AD or mood stabilizers, other forms of arrhythmias were studied less intensively.

Summaries of product characteristics (SmPCs) are product information for professionals [6]. The SmPC is a legal document applied as a feature of each medicine’s marketing authorization [7]. It gives healthcare professionals data on how to use the drug and is updated throughout the life cycle of the product as new data emerge [7]. The adverse drug reactions reported in the SmPCs should also contain input from spontaneous reporting (after careful checking the causal relationship between the drug and the adverse effect) [7]. The adverse effects are listed in section 4.8 of the SmPCs and are accessible in the European Medicines Agency (EMA) or the National Competent Authorities [7, 8]. The SmPCs estimate the frequency of adverse reactions based on the data source [9]. This data source is clinical trials, post-authorization safety studies, or spontaneous reporting, including causality evaluation [9]. In case the choice of the frequency category is based on different sources, the category representing the highest frequency is chosen unless a more specific method with clearly higher validity has been applied, e.g., a pooled analysis across suitable studies [9]. Sources of data use population exposed to the doses and treatment duration as recommended in the SmPC [9]. Adverse reactions are also derived from clinical trials, pooled placebo-controlled studies if sufficiently large to be informative, from active-controlled data, or possibly single-arm or add-on trial databases [9]. Frequency represents crude incidence rates and not differences or relative risks calculated against placebo or other comparators [9]. The adverse reactions are also derived from safety studies, in which the selection of the appropriate frequency category is based on the estimation of the crude incidence rate using standard statistical methods [9]. In concern to the adverse events reported from the spontaneous reporting system, each study where this adverse reaction could have been detected is reviewed to choose an adequate frequency category [9].

The current study addresses whether the frequency and, to some extension, the risk range of particular CAE (QT prolongation, Torsade de Pointes tachycardia, and ventricular arrhythmias) could be drawn from the information contained in the SmPCs. Only original medicinal products (OMP) of two essential classes of psychiatric drugs, namely AD and AP, were considered, especially, since it is assumed that approval studies on the OMP were more extensive and more accurate in quantifying adverse events than those for generics. Furthermore, excluding generic products allowed elimination of bioequivalence as a potential confounding factor for the risk assessment. On the other hand, generics arriving on the market are not subject of newly clinical studies regarding adverse effects. It has to be emphasized that creating a comprehensive risk assessment of all AD and AP regarding their potential to induce cardiac arrhythmias is beyond the scope of this study. Our aim was to address the arrhythmia risk of the psychotropic agents by reporting the respective frequency category mentioned in the SmPCs and categorize them accordingly.

## Methods

### Identification of active substances and original medicinal products

Data collection took place in March 2020. We used the anatomical-therapeutic-chemical (ATC) classification (group ATC: nervous system [N]) of the German Institute of Medical Documentation and Information (DIMDI) for the selection of the active substances of AP (N05A) and AD (N06A) [10]. As this database for medical documentation also contains active substances that are not approved in Germany, we conducted a further search for active substances on the “PharmaNet.bund-Portal,” which contains all approved substances in Germany. Since this online portal contains active substances for the treatment of humans and animals, the search had to be conducted as follows: using the name of the respective active substances (search category “substance name”) and using the corresponding search filtering criteria (“marketable human medicinal product”). We documented all active substances found using these criteria for a further detailed search for OMP.

We identified the OMP of AP and AD through the reporting data from the European medical agency (EMA) and through contact with each German marketing authorization holder for nationally authorized medicines (as described below). Once all OMP were identified, the correspondent SmPCs were collected from the online platforms “PharmNet.Bund” (www.pharmnet.bund), “Fachinfo service” (www.fachinfo.de), Rote Liste® (www.roteliste.de), and “Gelbe Liste®” (www.gelbe-liste.de/wirkstoffe/), and via manufactures. The SmPCs are legal documents considered as part of the marketing authorization of each product and provide precisely relevant information for healthcare professionals about the product. Updating of the SmPCs occurs throughout the life cycle of the drug [11]. In a second step, we analyzed the SmPCs of OMP of AP and AD and created a risk assessment of them, as explained below.

### Data collection and extraction

**Fig. 1:**
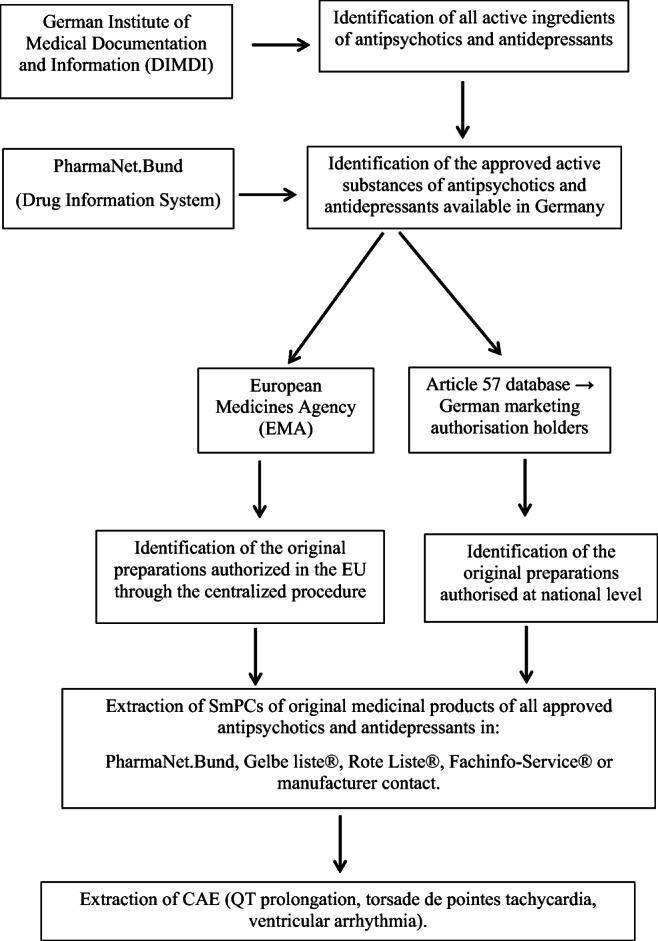
Flow diagram showing study design and procedures

To identify active substances as OMP and to differentiate them from generics, we downloaded the list of all the medicines authorized in the European Union (EU) through the centralized procedure on the EMA (https://www.ema.europa.eu/en/medicines/download-medicine-data). However, the authorization of products for the treatment of psychiatric conditions in Germany is regulated at the national level. Since the EMA does not differentiate registered active substances in originator medicines (OMP) versus generic medicines for nationally authorized products, we used the Article 57 database to identify OMP (Article 57 database lists medicines authorized nationally and centrally in the EU, but this database does not explicitly contain information on OMP). For each active substance of AD and AP that is listed in the Article 57 database and is nationally authorized, we contacted the German marketing authorization holder and asked for the name of the OMP of the active substance. Contacting at least one German marketing authorization holder for each active substance, we identified the name of all OMP of AD and AP approved in Germany (flow-chart for the search strategy for active substances and OMP is shown in Fig. 1).

In a second step, we performed first a search for the SmPC of those OMP on the PharmNet.Bund-Portal [12]. In case that we did not find the corresponding SmPC there, we searched further on the www.fachinfo.deportal [13], the www.rote-liste.deportal [14], and the www.gelbe-liste.deportal [15], respectively. If the SmPCs missed in all four previously mentioned online portals, the manufacturer was contacted and requested to send the corresponding SmPCs.

We documented the collected SmPCs of the OMP of both substance groups (AP and AD) in a master table. After listing all identified OMP, CAE of each original product was extracted from section 4.8 in the SmPC (section 4.8 in the SmPC reports about potential side effects in either comparison tables or lists according to the frequency rates of appearance). If CAE were reported in the SmPC, they were clustered in different ranges of appearance, ranging from “very common,” “common,” “uncommon,” “rare,” “very rare,” and “unknown” (as explained in Table 1). We focused on the most clinically relevant CAE (QT prolongation, Torsade de Pointes tachycardia, ventricular arrhythmia) and created ranking lists for AD and AP concerning the reported frequency of appearance. If the SmPC of a certain OMP did not mention any of these three CAE, this OMP was not added to the ranking list. Furthermore, we documented and counted the total number of reported CAE (all kinds of CAE) in the SmPC of each OMP (of AD and AP), and tabulated the data. To keep the data as structured as possible, we implemented inclusion and exclusion criteria (Table 2).

**Table 1:** Frequencies of side effects were featured using the intervals indicated in the section 4.8 in the German summary of product characteristic (SmPC)

German	English	Frequency	Color
“Sehr häufig”	“Very common”	≥ 1/10	Red
“häufig”	“Common”	≥ 1/100–< 1/10	Orange
“gelegentlich”	“Uncommon”	≥ 1/1.000–< 1/100	Yellow
“selten”	“Rare”	≥ 1/10.000–< 1/1.000	Light orange
“sehr selten”	“Very rare”	≤ 1/10.000	Light blue
“nicht bekannt”	“Not known”	?	Gray

**Table 2:** Inclusion and exclusion criteria

Inclusion criteria	Exclusion criteria
Active substances of antipsychotics and antidepressants that were found on the ATC index and were also found in the search mentioned above (criteria of PharmNet.Bund-portal).	Active substances of antipsychotics and antidepressants that were found on the ATC index but missed in the search mentioned above (criteria of PharmNet.Bund-portal).
Whenever possible, only the oral application form (tablets, dragees, and capsules).	Other forms of application (e.g., solutions for Injection) or depot-preparations*, except if this form of application is the only available form for an OMP.^†^
The SmPC with the highest dose approved for adult psychiatry practice.	OMP that are either withdrawn or removed from the Market, or discontinued from the sale, or not produced anymore, or unavailable in the Market, or non-availability of an SmPC, or when the manufactured was not reached**
Only currently available PM in the German market	
Pure St. John’s wort.	St. John’s wort combined with other herbal substances.

*Depot preparations, such as Trevicta® (paliperidone), Olanzapine® Zypadehra® (olanzapine), and Abilify maintena® (aripiprazole), were also excluded

^†^Dapotum® (fluphenazine) and IMAP® (fluspirilen) were included despite being in intramuscular injection form. We also added Xomolix® (droperidol), as it is only available in the form of an injection solution. We included Adasuve® (loxapine), which is only available as inhalation. We included Spravato® (esketamine), which is only available as a nasal spray. We also included Tiaprid® (tiapride), a benzamide (Antipsychotics Group, N05AL), which in Germany has no authorization for the treatment of mental disorders, but antipsychotics-induced late dyskinesia

**An example for AD and AP, which were withdrawn from the German market and therefore excluded from the study, are lurasidone, sibutramine, desipramine, nefazodone, perphenazine

**We excluded Decentan® (perphenacine), withdrawal since 01. February 2013. We also excluded Fluctin® (fluoxetine), as Lilly Germany GmbH (manufacturer) informed us via E-mail about its discontinuation from sale due to the availability of plenty of generics. We also excluded Tofranil® (imipramine), as the manufacturer informed us via E-mail that it is not available in the market for many years. We excluded Truxal® (chlorprothixene), as Lundbeck informed us that Truxal was removed from the German market. We excluded Taxilan® (perazine), as Taxilan was also removed from the German market. We also excluded Tolvin® (mianserin), as we did not find an SmPC in the four previously mentioned online portals, and we were not able to contact the manufacturer. Thombran® (trazodone) was excluded for the same reasons. We excluded Equilibrin® (amitriptylinoxide), as the manufacturer Sanofi informed us via E-mail that they do not produce it anymore. Dapotum® (fluphenazine) was also excluded due to the non-availability of a SmPC

## Results

**Fig. 2:**
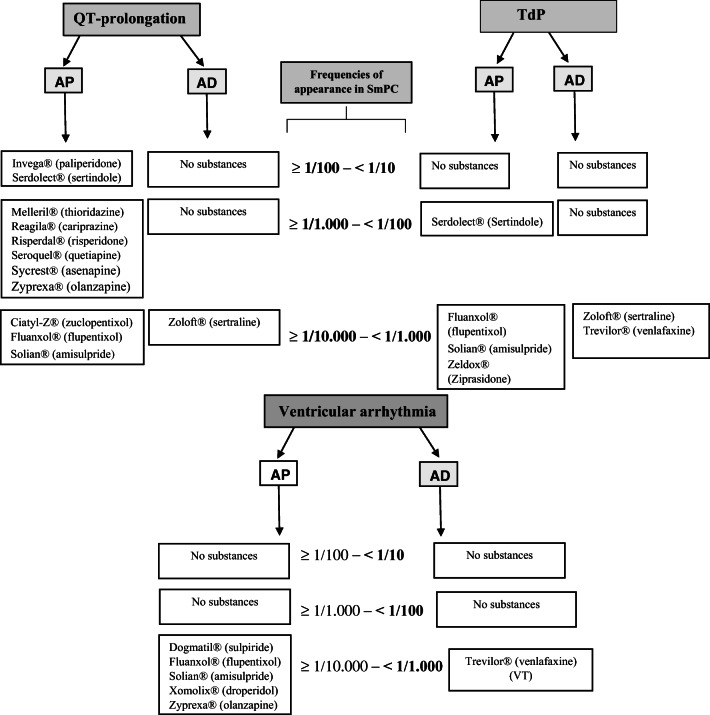
A diagram presenting original medical products (OMP) of antipsychotics (AP) and antidepressants (AD) that are associated with cardiac adverse events (CAE). TdP, Torsade de Pointes; VT, ventricular tachycardia

For identifying OMP of AP and AD authorized in Germany, it was necessary to search in different sources (EMA, Article 57 database, DIMDI, PharmaNet.bund-Portal, Fachinfo service, Rote Liste®, and Gelbe Liste®) and to directly contact different manufacturers. We identified 50 OMP with 26 SmPCs of AP and 24 SmPCs of AD (Fig. 2).

### Risk of QT prolongation

According to the SmPCs, Invega® (paliperidone) and Serdolect® (sertindole) had a comparably higher risk of QT prolongation among both AP and AD (≥ 1/100 and < 1/10). Zyprexa® (olanzapine), Reagila® (cariprazine), Risperdal® (risperidone), Seroquel® (quetiapine), Sycrest® (asenapine), and Melleril® (thioridazine) were all associated with the frequency range risk category (≥ 1/1000 and < 1/100). Solian® (amisulpride), Ciatyl-Z® (zuclopenthixol), Fluanxol® (flupentixol), and among AD Zoloft® (sertraline) were all associated with the following frequency range category (≥ 1/10.000 and < 1/1000).

### Risk of torsade de pointes

Regarding TdP, Serdolect® (sertindole) was associated with following frequency range risk category (≥ 1/1000 and < 1/100). Solian® (amisulpride), Zeldox® (ziprasidone), Fluanxol® (flupentixol), and among AD, Zoloft® (sertraline) and Trevilor® (venlafaxine) were all associated with the frequency risk category (≥ 1/10.000 and < 1/1000). Atosil® (promethazine), Melleril® (thioridazine), Xomolix® (droperidol), Neurocil® (levomepromazine), and among AD, Saroten® (amitriptyline), Anafranil® (clomipramine), and Aponal® (doxepin) were all associated with the frequency range risk category (< 1/10.000).

### Risk of ventricular arrhythmia

Regarding ventricular arrhythmia, Solian® (amisulpride), Xomolix® (droperidol), Zyprexa® (olanzapine) (ventricular tachycardia), and Fluanxol® (flupentixol), Dogmatil® (sulpiride) were all associated with the frequency risk category (≥ 1/10.000 and < 1/1000), while among AD, Trevilor® (venlafaxine) was associated with (≥ 1/10.000 and < 1/1000)) risk for ventricular tachycardia (Table 3). The created rank whole-list resulted from the frequencies of appearance of the mentioned three CAE in the SmPC of the OMP of AP, and AD is shown in Table 3.

**Table 3:** The risk of QT prolongation, Torsade de pointes (TdP), and ventricular arrhythmia related to original products of antipsychotics and antidepressants according to the German summaries of product characteristics (SmPC) (brand name and active substance name are reported)

Frequency	QT prolongation	TdP	Ventricular arrhythmia
“Very common” (≥ 1/10)			
“Common (frequent)” (≥ 1/100 and < 1/10)	Invega® (paliperidone), Serdolect® (sertindole)		
“Uncommon (infrequent)” (≥ 1/1000 to < 1/100)	Zyprexa® (olanzapine), Reagila® (cariprazine), Risperdal® (risperidone), Seroquel® (quetiapine), Sycrest® (asenapine), Melleril® (thioridazine)	Serdolect® (sertindole)	
“Rare” (≥ 1/10.000 and < 1/1000)	Solian® (amisulpride), Ciatyl-Z® (zuclopentixol), Fluanxol® (flupentixol), Zoloft® (sertraline)	Solian® (amisulpride), Zeldox® (ziprasidone), Fluanxol® (flupentixol), Zoloft® (sertraline), Trevilor® (venlafaxine)	Solian® (amisulpride), Xomolix® (droperidol), Zyprexa® (olanzapine), (ventricular tachycardia), Fluanxol® (flupentixol), Dogmatil® (sulpiride), Trevilor® (venlafaxine) (ventricular tachycardia)
“Very rare” (< 1/10.000)	Xomolix® (droperidol), Anafranil® (clomipramine)	Atosil® (promethazine), Melleril® (thioridazine), Xomolix® (droperidol), Neurocil® (levomepromazine), Saroten® (amitriptyline), Anafranil® (clomipramine), Aponal® (doxepin)	
“Frequency unknown” (= risik is unclear)*	Atosil® (promethazine), Glianimon® (benperidol), Eunerpan® (melperone), Dogmatil® (sulpiride), Tiapridex® (tiapride), Stangyl® (trimipramine), Aponal® (doxepin), Tolvin® (mianserin), Cipramil® (citalopram), Cipralex® (escitalopram),	Haldol® (haloperidol), Abilify® (aripiprazole), ORAP® (pimozide), Eunerpan® (melperone), Dogmatil® (sulpiride), Tiapridex® (tiapride), Stangyl® (trimipramine), Tolvin® (mianserin), Cipramil® (citalopram), Cipralex® (escitalopram)	Haldol® (haloperidol), (ventricular tachycardia), Abilify® (aripiprazole), ORAP® (pimozide), (ventricular tachycardia), (ventricular tachycardia), Eunerpan® (melperone), (ventricular tachycardia), Glianimon® (benperidol), Tiapridex® (tiapride), Tolvin® (mianserin), Cipramil® (citalopram), Cipralex® (escitalopram)

### The absolute number of reported CAE (all kinds of CAE)

Leponex® (clozapine) was among all AP associated with the highest number of CAE (16 CAE), while among AD, Anafranil® (clomipramine) was associated with the highest number of CAE (12 CAE). Each of the following Tables (Table 4 and Table 5) classifies the original products of the identified 50 OMP in different categories according to the number of reported CAE.

**Table 4:** The maximal number of reported CAE related to antipsychotics (original products) according to the German summaries of product characteristics (SmPC)

Original product (active substance)	Number of CAE
Abilify® (aripiprazole)	6
Adasuve® (loxapine)	0
Atosil® (promethazine)	5
Ciatyl-Z® (zuclopentixol)	4
Dogmatil® (sulpiride)	8
Dominal® (prothipendyl)	5
Eunerpan® (melperone)	8
Fluanxol® (flupentixol)	5
Glianimon® (benperidol)	5
Haldol® (haloperidol)	5
IMAP® (fluspirilen)	1
Invega® (paliperidone)	10
Leponex® (clozapine), Elcrit (clozapine)	16
Melleril® (thioridazine)	6
Neurocil® (levomepromazine)	6
ORAP® (pimozide)	3
Reagila® (cariprazine)	5
Risperdal® (risperidone)	9
Serdolect® (sertindole)	3
Seroquel® (quetiapine)	5
Solian® (amisulpride)	8
Syncrest® (asenapine)	4
Tiapridex® (tiapride)	7
Xomolix® (droperidol)	7
Zeldox® (ziprasidone)	3
Zyprexa® (olanzapine)	5

**Table 5:** The maximal number of reported CAE related to antidepressants according to the German summaries of product characteristics (SmPC)

Original product (active substance)	Number of CAE
Anafranil® (clomipramine)	12
Aponal® (doxepin)	7
Aurorix® (moclobemide)	0
Brintellix® (vortioxetine)	0
Cipralex® (escitalopram)	5
Cipramil® (citalopram)	6
Cymbalta® (duloxetine)	4
Edronax® (reboxetine)	2
Elontril® (bupropion)	2
Fevarin® (fluvoxamine)	2
Hyperforat® (St. John’s wort)	0
Insidon® (opipramole)	7
Jatrosom N® (tranylcypromine)	1
Ludiomil® (maprotilin)	10
Milnaneurax® (milnacipran)	8
Remergil® (mirtazapine)	0
Saroten® (amitriptyline)	10
Seroxat® (paroxetine)	2
Spravato® (esketamine)	1
Stangyl® (trimipramine)	11
Tianeurax® (tianeptine)	3
Trevilor® (venlafaxine)	8
Valdoxan® (agomelatine)	0
Zoloft® (sertraline)	7

## Discussion

Our study is the first to use data extracted from the SmPCs to rank the AD and AP according to their risk to induce CAE. To make a uniform comparison, we decided to consider only the original brand name product (i.e., no generics) of both AD and AP, which are available in the German market. The identification of the approved brand name drugs in Germany was challenging due to the absence of a database containing OMP.

According to the analysis of SmPCs, Serdolect® (sertindole), Invega® (paliperidone), and Solian® (amisulpride) were the most critical AP concerning the risk of cardiac arrhythmias. These substances are effective AP so that their replacement might be associated with a risk of ineffectiveness or intolerance [16]. It is well-known that sertindole belongs to the drugs with high risk of cardiac arrhythmias, and was therefore temporarily suspended from the European market in the nineties [17]. Although our analysis put sertindole in its corresponding place as a high-risk drug, other drugs were found to be in a similar risk category, a magnitude that has not been observed trough previously published data until now.

In a previous study, the risk of cardiac arrhythmia related to the use of psychiatric drugs has been estimated based on data from different sources, including EMA, FDA, as well as Micromedex, CredibleMeds.org, and the Maudsley prescribing guideline [18]. Based on an analysis of data from these sources, the authors classified the psychiatric drugs into three categories according to their risk to induce cardiac arrhythmias [18]**.** The category with the highest risk included neither paliperidone nor amisulpride, and agents like haloperidol, ziprasidone, and pimozide fell into this category. However, the study of Fanoe et al. differs from our study in different aspects. First, we used SmPcs of only brand name drugs as the sole source of information. Whether the inclusion of generic drugs has affected the results still needs to be investigated. Our categorization was not based on the overall risk of inducing cardiac arrhythmias; instead, we studied more detailed distinct forms of arrhythmias, i.e., QT prolongation, TdP, or ventricular tachycardia. Furthermore, we included oral formulations of the drugs, with few exceptions, as explained in the methods section. This might explain why, e.g., haloperidol was not among the drugs with the highest risk based on our analysis, because this risk is mostly related to the intravenous application of the drug [19]**.** Several other studies have also demonstrated that haloperidol is a critical drug regarding its potential to induce severe forms of cardiac arrhythmias even after oral application. However, these studies have been performed in elderly or critically ill patients [20–22]. Our risk categorization based on analysis of SmPCs for the population level for whom the substance is indicated (age independent), while the elderly patients are considered a specific patient group, which needs specific information.

Regarding AD, our findings have shown that Zoloft® (sertraline), Trevilor® (venlafaxine), and Anafranil® (clomipramine) appear to be critical substances concerning the risk of cardiac arrhythmias. Our results differ from those of Fanoe et al., which were based on a different approach. According to their risk categorization, neither venlafaxine nor sertraline falls into the highest risk category. In this study, sertraline was even considered to be without any risk of QT prolongation or TdP.

The risk assessment of cardiac arrhythmia of AD has also been investigated in a large Swedish register study [22]. In this study, the authors used a different approach to estimate the risk of TdP, where they linked death outside the hospital and TdP associated with the use of psychotropic drugs after adjusting for comorbidity and several other confounders [22]. They included all persons aged 65 or over in Sweden who died outside hospitals from 20,082,013 (*n* = 286,092) and matched controls [22]. According to their analysis, mirtazapine, citalopram, sertraline, and amitriptyline were the ones associated with the highest risk of mortality [22]. Here, it is worth noting that this study was a register study, and this conclusion was based on statistical analysis and not an actual observation. Moreover, the study included only a subset of the population, namely, those aged over 65, so the results might not reflect the risk within the whole population.

In 2020, Danielsson et al. performed a prospective register-based cohort study in Sweden, in which TdP events in association with the use of drugs labeled with TdP risk were examined in two age groups, 18 to 65 and the above 65 age group [23]. Danielsson et al. concluded that TdP is more frequent in the geriatric population than in younger age groups [23]. According to Danielsson et al., citalopram had at least double the incidence of TdP per 100 thousand patients during the study period, a trend that was observed in the geriatric population and also in the younger age group [23]*.* We could not retrieve the results of Danielsson et al. in our study; in our risk assessment, citalopram was associated with an unknown risk of TdP. However, a significant limitation of Danielsson et al.’s study was that information about drug treatment during hospital stay was not available in the Swedish Prescribed Drug Register, and it was challenging to prove actual drug compliance; those are confounding factors that limit providing valid data for assessment [23]*.* In comparison, information about drug treatment during hospital stay should not be missed in the SmPCs, as physicians are legally obliged to report adverse events during hospital stay [23]*.* However, the register-based approach of Danielsson et al. [23] and also our approach based on the SmPCs reflect the difficulty of providing valid data to study rare adverse events.

Our analysis ensured a uniform comparison of AD and AP regarding their risk to induce different forms of CAE since we used the same source of information for all drugs, and we included only the brand name drugs. However, our study may have some limitations. Previously, the SmPCs have been criticized for contained incomplete information [24, 25], and SmPCs might also not be regularly updated after first approval, which means that CAE reported through post-marketing surveillance might be missing (at least for a certain time) [26]. Moreover, collecting data from SmPCs implies some difficulties; for example, different synonyms have been used to describe the same side effect. For example, the terms “ventricular tachycardia” and “ventricular tachyarrhythmias” describe the abnormal ventricular frequency; both terms may indicate different meanings. This might be due to the lack of uniform coding while reporting adverse effects in clinical trials [27]. Another example of difficulties in extracting data from SmPCs is the uncertainty in reporting the frequency category of adverse effects. One of the frequency categories is designated to be “unknown,” which means that frequency cannot be estimated from available data. This category cannot be translated to numerical information in the risk assessment. Although some drugs such as citalopram and escitalopram showed a risk to induce cardiac arrhythmia in several studies [28], their risk category in the SmPCs was reported to be unknown.

Assessing the risk of CAE associated with the use of psychiatric drugs is of outmost clinical importance. The availability of a reliable tool reporting such risks might be helpful to the prescribing physician. Our study has created rank lists (category assessment) of AD and AP based on their potential to induce different types of arrhythmias according to the analysis of the SmPCs, including unusually severe or life-threatening arrhythmias. Based on our analysis of SmPCs, Adasuve® (loxapine) carries the lowest cardiac risk, while Serdolect® (sertindole) carries the highest risk of CAE. Regarding AD, Brintellix® (vortioxetine) carries the lowest cardiac risk, while Zoloft® (sertraline), Trevilor ® (venlafaxine), and Anafranil ® (clomipramine) carry the highest risk of CAE.

Although SmPCs represent an important source of information for safety issues and prescribing decisions (they are the official and essential source of information for healthcare professionals), much room remains for improvement and future work in this respect. SmPC information are the result of the approval studies and must be regularly updated by new post-marketing observations on CAE through the surveillance spontaneous reporting systems. Pharmaceutical companies, consultants, and pharmacists are legally obliged to report adverse events [29]. All messages are collected and evaluated within the European EudraVigilance database and are regularly evaluated at intervals of 2 or 4 weeks [30]. The Pharmacovigilance Risk Assessment Committee (PRAC) evaluates signals from EudraVigilance and might recommend appropriate regulatory measures, including updating the informing texts [31]. Nevertheless, occasionally until now, unknown CAE appear for the first time after post-marketing experiences and these CAE (or changes in the observed frequency) are not immediately adopted in the SmPC. Therefore, the SmPC enables only a limited quantitative risk assessment. That is why results from analysis of the SmPCs should also be combined with other sources of data such as placebo-controlled single-substance studies, head-to-head studies, or meta-analysis assessing the effect of psychotropic drugs directly on cardiac rhythm.

## Conclusion

Categorization of the psychotropic drugs with concern to the risk of cardiac arrhythmia is of extreme clinical importance. The utilization of SmPCs for assessing cardiac risks based on the frequency categories of the CAE might aid in selecting drugs carrying a lower risk of CAE. This targeted way of using the SmPC for a risk evaluation is only possible if the information contained in it from certain substance classes is contrasted and evaluated, as we have done in the presented work. However, the interpretation of reported risk assessment has to be done carefully, considering the limitations of SmPCs.

## Data Availability

Data will be available on request by the authors.
